# The significance of green innovation efficiency of green low-carbon circular economy for sustainable cities in western China—Empirical evidence from Chongqing municipality

**DOI:** 10.3389/fpubh.2025.1617297

**Published:** 2025-07-11

**Authors:** Panfeng Li

**Affiliations:** Business School, Zhengzhou University of Industrial Technology, Zhengzhou, China

**Keywords:** green low-carbon circular economy, green innovation efficiency, sustainable city, entropy weight-TOPSIS, panel regression modeling

## Abstract

**Introduction:**

The Green Low-Carbon Circular Economy (GLCCE) represents a critical pathway toward achieving sustainable development, particularly within the context of ongoing urbanization in western China. This study investigates the role of open innovation, specifically through green innovation efficiency (GIE), in advancing GLCCE and fostering sustainable urban development. Chongqing Municipality serves as an empirical case, utilizing data from 2014 to 2023.

**Methods:**

An evaluation indicator system for GLCCE was established, encompassing dimensions of economic and social development, green development, low-carbon development, and circular development. The entropy weight-Technique for Order Preference by Similarity to Ideal Solution (TOPSIS) model was employed to calculate Chongqing's GLCCE composite index. Subsequently, a panel regression model was developed to assess the impact of green innovation efficiency (GIE) on environmental quality. In this model, SO_2_ concentration was the dependent variable, GIE was the core explanatory variable, and control variables included openness to external trade (EXT), information technology level (ITL), urban cultural level (CUL), and research and development investment (RDI).

**Results:**

The entropy weight-TOPSIS model revealed that Chongqing's GLCCE composite index increased from 0.405 in 2014 to 0.684 in 2023, with a peak of 0.866 in 2020, indicating significant overall progress in GLCCE implementation. The panel regression analysis demonstrated that GIE significantly reduces SO_2_ concentration, with a coefficient of −0.218 (*p* < 0.05). This signifies that a 1% increase in GIE is associated with a 0.218-unit decrease in SO_2_ concentration, underscoring GIE's role in improving environmental quality through technological advancement and resource optimization.

**Discussion:**

The findings highlight substantial advancements in Chongqing's GLCCE and underscore the pivotal contribution of green innovation efficiency to this progress, particularly in enhancing environmental quality. The significant negative relationship between GIE and SO_2_ concentration suggests that fostering innovation is crucial for urban sustainability and improving living standards. This study provides empirical evidence and offers valuable policy insights for other cities in western China aiming to promote sustainable development through the GLCCE framework and strategic open innovation initiatives.

## 1 Introduction

Against the backdrop of global climate change, resource scarcity, and increasing environmental pollution, the Green Low-Carbon Circular Economy (GLCCE) has emerged as a pivotal pathway for achieving the United Nations 2030 Sustainable Development Goals (SDGs) (1). The Paris Agreement and the Glasgow Convention on Climate Change underscore the necessity for nations to decouple economic growth from carbon emissions through technological innovation and industrial transformation (2). Within this context, the Circular Economy (CE) model (3) enhances resource efficiency and minimizes environmental impact via the “Reduce, Reuse, and Recycle” principles. Concurrently, the Low-Carbon Economy (LCE) (4) prioritizes adjustments in energy structures and the application of carbon reduction technologies, while the Green Economy (GE) (5) seeks to harmonize ecological preservation with economic advancement. The GLCCE integrates these paradigms by promoting economic activities that simultaneously embody the waste reduction and resource looping of CE, the emission control of LCE, and the biodiversity and ecosystem protection of GE. For example, in urban manufacturing, GLCCE encourages the use of recycled materials, renewable energy, and environmentally responsible supply chains, thereby addressing multiple sustainability dimensions. This integrated approach is particularly vital for western China, where balancing economic growth with ecological preservation is critical due to resource constraints and environmental vulnerabilities. As such, GLCCE has become a core strategy for sustainable urban development worldwide, with significant implications for regions like western China.

As the world's largest developing country, China faces the double pressure of economic development and environmental protection (6). For this reason, China has put forward a “dual-carbon” strategic goal, i.e., carbon peaking and carbon neutrality, in order to promote a comprehensive green transformation of economic and social development (7). The western region of China is an important ecological security barrier and economic development potential area in China (8). The western region has rich natural resources and diverse ecosystems, but also faces problems such as relatively low level of economic development (9) and unreasonable industrial structure (10). Western China (e.g., Chongqing, Sichuan, Shaanxi, etc.) occupies an important position in the national “Western Development” and “Yangtze River Economic Belt” strategies, but faces the contradiction between economic growth and ecological protection. The main manifestations are: (1) Resource-dependent economy: some cities are still dominated by heavy industries with high energy consumption and high emissions (e.g., iron and steel, chemical industry), which leads to low energy utilization efficiency and high pressure of environmental pollution (11). (2) Ecological vulnerability: the western region is an important ecological barrier in the upper reaches of the Yangtze River and the Yellow River, but the problems of soil and water erosion and rocky desertification are prominent (12). (3) Insufficient resources for innovation: compared with the eastern coastal region, the western region has deficiencies in green technology research and development (13), high-end talent reserves (14), and the introduction of international capital (15).

GIE (16), as the core driving force of the GLCCE, has become the core grip to crack the development dilemma in the west. GIE refers to the process of enhancing efficiency through technological innovation and optimal resource allocation. It aims to achieve synergy between environmental protection and economic growth (17). GIE not only involves technology research and development and application, but also includes technology diffusion, industrial structure upgrading and comprehensive improvement of resource utilization efficiency. Studies have shown that GIE can significantly improve the efficiency of circular economy and reduce environmental pollution through technological progress and optimal allocation of resources, thus providing important support for sustainable development (16). Globally, GIE has become an important factor in promoting the high-quality development of GLCCE, especially in resource-dependent economy and ecologically fragile areas.

The specific mechanisms through which GIE drives GLCCE can be categorized into four key pathways: (1) Technological innovation: GIE enhances the efficiency of resource utilization and pollution control through the development and adoption of green technologies such as industrial desulfurization and carbon capture, directly reducing environmental externalities; (2) Structural transformation: by fostering innovation, GIE facilitates the shift from heavy, resource-intensive industries to high-tech and service-oriented sectors, thus promoting an industrial structure more aligned with circular economy principles; (3) Knowledge diffusion: GIE supports the dissemination of environmentally friendly technologies and best practices through open innovation networks, including external collaborations and imported patents; and (4) Policy and investment efficiency: high GIE levels attract clean-tech investments and enable more effective implementation of green fiscal and regulatory policies. These mechanisms synergistically contribute to GLCCE by reducing pollutant emissions, improving energy productivity, and enabling sustainable urban development.

As an important city in western China, Chongqing Municipality has a typical representative and important strategic position. Chongqing Municipality was selected as the empirical case for this study due to its strategic importance, unique characteristics, and representativeness within western China. As the only direct-controlled municipality in the region, Chongqing serves as a critical hub for green development along the Yangtze River Economic Belt. Its topography, characterized by mountainous terrain and a strategic river confluence, presents unique ecological challenges and opportunities for sustainable development. Industrially, Chongqing is a manufacturing powerhouse, with sectors like automotive and electronics driving its economy, making it a representative case for studying the transition from traditional industries to a green, low-carbon circular economy. Additionally, Chongqing benefits from national policy support, such as its inclusion in the Chengdu-Chongqing economic circle and the Yangtze River Economic Belt, which provide it with development advantages that are emblematic of broader regional strategies. While Chongqing's specific outcomes may not be fully generalizable across all western cities due to its distinct features, it serves as a model for how cities in ecologically sensitive and industrially diverse regions can leverage green innovation to achieve sustainable development. We focused on Chongqing to provide an in-depth, data-driven analysis, leveraging its comprehensive statistical records and clear policy framework, which enabled robust empirical modeling.

In recent years, Chongqing Municipality has actively explored the development of GLCCE and achieved certain results. Therefore, this study takes Chongqing Municipality as an empirical case to explore how GIE affects the development of GLCCE and further promotes the construction of sustainable cities. Open innovation, in the context of this study, refers to the strategic use of external knowledge, technologies, and collaborations to enhance green innovation efficiency, as conceptualized by Sá et al. (18). In green innovation, open innovation involves adopting externally developed green technologies and establishing collaborative R&D networks through partnerships with firms, research institutions, and governments to co-develop sustainable solutions. These dimensions are critical for GLCCE, as they facilitate the rapid diffusion and implementation of environmentally friendly technologies. In our analysis, open innovation is reflected through the GIE variable, which integrates internal innovation capabilities with the benefits of external collaboration, as detailed in the Section 3.

The innovations and unique contributions of this study are as follows: (1) Introduced open innovation metrics (external tech adoption, collaborative R&D networks) into GIE measurement. (2) Proposed the hybrid entropy-weighted TOPSIS model to dynamically quantify GLCCE development levels, overcoming limitations of static or single-method approaches. (3) Integrating open innovation, which leverages external knowledge, technologies, and collaborative R&D networks, into the promotion of the GLCCE.

## 2 State of the art

### 2.1 Concept definition

As an innovative economic development model, the core of GLCCE lies in the systematic integration of environmental factors and economic growth objectives (19). This model breaks through the one-way path dependence of the traditional linear economy of “resource extraction-processing and production-product disposal,” and realizes the stepwise utilization and recycling of waste through the construction of the closed-loop feedback process of “resource-product-recycled resource.” This non-linear paradigm reconstructs the synergistic relationship between economic development and resource utilization, and significantly reduces environmental externality costs while enhancing resource metabolism efficiency. At present, the academic community has not yet formed a unified consensus on the precise concept of GLCCE. Scholars in various fields have interpreted GLCCE from different perspectives according to their research needs. However, in general, there is a consensus in the academic community that the circular economy takes green development as its core and realizes the unity and maximization of ecological, economic and social benefits through the efficient use of resources. In summary, the study adopts the concept of circular economy agreed upon by most scholars. GLCCE is centered on the efficient use of resources and recycling, following the principle of “Reduce-Reuse-Recycle” (20), with low consumption, low emissions, high efficiency, in line with the concept of sustainable development. The concept of “3R” principle as the core, a more complete description of the connotation and key aspects of the circular economy, for the implementation of the circular economy points out the direction.

### 2.2 Research status

The current academic research on “GLCCE system” mainly focuses on the following aspects: First, the theoretical research on GLCCE system. Based on the theoretical framework of industrial ecology, Du et al. (21) define China's GLCCE system as: reconstructing the metabolic mechanism of the economic system with ecological rationality, and realizing the development transformation through endogenous driving path. The core of the system is to follow the law of ecosystem material cycling to achieve synergistic gains in economic and ecological wellbeing; to systematically resolve the coupling of economic growth with environmental pressure and resource constraints, and to complete the structural change of the development paradigm. In terms of the realization path, Zhu and Li (22), in view of the common problems of modern urban governance, believe that the country should effectively optimize the urban GLCCE system through the establishment of a scientific decision-making mechanism. Duan et al. (23) posit that accelerating the refinement of fiscal, taxation, and financial systems for green low-carbon circular economies, coupled with the implementation of clean energy industrial policies, can effectively facilitate the attainment of carbon peak and carbon neutrality objectives. These strategic measures ultimately contribute to establishing a comprehensive framework for an all-encompassing green low-carbon circular economic system. The theory of “Performance Economy” put forward by Xie et al. (24) emphasizes the restructuring of the value creation model from the perspective of the life cycle of the product. Their research shows that adopting the circular economy model can increase the resource productivity of the industrial system by more than 30%. Talla and McIlwaine (25) released the “Action Guide for Circular Economy,” which systematically elaborates the whole-chain transformation path of “design-production-consumption-recycling,” and in particular points out the key role of digital technology in the tracking of material flow.

Secondly, it is a study on the measurement of GLCCE system. Xin et al. (26) constructed an index system based on four levels of development power, production system, living system and development benefits, and used the spatio-temporal extreme difference entropy weight method to measure the level of construction of GLCCE development system. The study found that the construction level of GLCCE system has been improved all over the country. Zhang et al. (27) constructed a GLCCE evaluation index system from the three dimensions of low-carbon, green, and circular, and measured the construction of provincial green low-carbon economic system by using a dynamic comprehensive measurement model. The results found that the overall development level of China's GLCCE system is relatively stable. International academic research on GLCCE is characterized by multidisciplinary cross-fertilization. Safarzynska et al. (28) used Dynamic Stochastic General Equilibrium (DSGE) model to simulate the impact of different carbon pricing mechanisms on the transition to a circular economy, and found that the policy combination of stepped carbon tax together with research and development subsidies can increase the speed of the transition by 40%. Subramaniam et al. (29) based on the panel data of the European Union 28 countries, and verified that the marginal effect of the extended producer responsibility system on the e-waste recycling rate is 0.15. This result provides a quantitative basis for policy formulation. Furthermore, empirical studies have applied these concepts to specific regions and contexts. For instance, Ren et al. (30) analyzed the implementation of circular economy practices in Chongqing, emphasizing the city's efforts in waste management and resource recycling as part of its GLCCE strategy. Additionally, research has established a positive correlation between green innovation efficiency and sustainable development outcomes. Hayat and Qingyu (17) demonstrated that green innovation strategies enhance sustainable innovative performance, underscoring the significance of GIE in driving sustainability.

Despite these advancements, the current research on GLCCE systems exhibits several limitations and gaps that warrant further exploration. First, there is a limited focus on regional heterogeneity. Many studies, such as Xin et al. (26) and Zhang et al. (27), adopt a national or provincial perspective, which often overlooks the unique ecological, economic, and industrial characteristics of specific regions like western China. For instance, the resource-dependent economies and ecological vulnerabilities of cities like Chongqing require tailored GLCCE strategies, yet few studies address these regional nuances. Second, there is insufficient integration of social and behavioral factors into GLCCE frameworks. While theoretical models like those of Du et al. (21) emphasize ecological and economic dimensions, the role of societal engagement—such as public environmental awareness or consumer behavior in waste reduction—is underexplored, limiting the understanding of GLCCE's social sustainability. Third, challenges in dynamic and long-term modeling persist. Current measurement methodologies, such as the entropy weight method used by Xin et al. (26), often rely on static or short-term data, which may not capture the evolving nature of GLCCE systems under external shocks like global energy crises or policy shifts. These gaps highlight the need for more region-specific, socially inclusive, and dynamic approaches to GLCCE research, which our study aims to address by focusing on Chongqing's unique context and integrating open innovation metrics.

## 3 Methodology

### 3.1 Study area and data sources

#### 3.1.1 Overview of the study area

Chongqing, situated in western China and spanning longitudes of 105°11′-110°11′ east and latitudes of 28°10′-32°13′ north, is the only direct-controlled municipality in the region and a critical hub for green development along the Yangtze River Economic Belt. Its geomorphological features show a significant “east-southwest hills.” The geomorphological features of the area show a significant “east-southwest hill” differentiation. The western and central parts of the city are part of the hilly area on the eastern edge of the Sichuan Basin, with an average elevation of 491 meters and a slope of ≤ 15° accounting for 62% of the area. This geography has led to the formation of a green manufacturing corridor centered on the automotive and electronics industries. The eastern region, on the other hand, straddles the Daba-Wuling mountain system, with an average elevation of 869 meters and a slope ≥25° accounting for 78% of the area, making it an important ecological product supply area in the country. The city's mountainous area accounts for 76.3% of the total (as of 2023), and its Ecological Vulnerability Index (EVI) is 0.82, which is significantly higher than the national average of 0.65, making it an ideal sample for studying the efficiency of green innovation for a GLCCE.

According to the Outline of the Plan for the Construction of the Chengdu-Chongqing Twin-city Economic Circle (2023), Chongqing has constructed a synergistic development pattern of “one region and two clusters.” This provides a platform for differentiated policy experimentation for a GLCCE. The Main City Metropolitan Area (covering 21 districts), as one of the country's first climate-resilient pilot cities, focuses on green technological innovation, with green patents authorized accounting for 12.3% of the western region in 2023, while the digital economy accounts for 45.7% of GDP. The Three Gorges Reservoir Area (11 districts and counties) in northeast Chongqing, the country's first inter-provincial ecological compensation pilot area, saw its Gross Ecological Product (GEP) exceed RMB 420 billion in 2023. And the Wuling Mountain Area (six districts and counties) in southeast Chongqing, as a national pilot area for comprehensive ecological compensation, has a forest coverage rate of 68.5% (in 2023) and accounts for 38% of the city's carbon sinks trading volume. This series of initiatives not only reflects Chongqing Municipality's balance between ecological protection and economic development, but also demonstrates its active exploration and remarkable results in promoting regional green and low-carbon development.

#### 3.1.2 Data sources and processing

This study examines Chongqing Municipality's GLCCE development level from 2014 to 2023. Data for the evaluation indicators were obtained from the China Statistical Yearbook, China Environmental Statistical Yearbook, China Energy Statistical Yearbook, Chongqing Statistical Yearbook, and various statistical bulletins published by Chongqing Municipality. Provincial carbon emission data were sourced from the China Emission Accounts Datasets (CEADs). Missing values in the indicators were addressed using methods such as substitution with similar indicators, linear regression estimation, and interpolation.

### 3.2 Construction of the evaluation indicators for GLCCE system

This study adopts a logical framework that includes four primary indicators: economic and social development, green development, low-carbon development, and circular development. Building on frameworks proposed by prior scholars, the study tailors the framework to Chongqing Municipality's specific development context and resource characteristics. Following the principles of scientificity, systematicness, and operability, 17 secondary indicators were selected to form the evaluation indicators for GLCCE system in Chongqing. The indicator system is presented in Table 1.

**Table 1:** Overview of the evaluation indicators for GLCCE system in Chongqing municipality.

**Primary indicators**	**Secondary indicators**	**Indicator symbol**	**Indicator type**
Economic and social development	Per capita GDP (10,000 yuan)	L1	Positive
	Proportion of the tertiary industry (%)	L2	Positive
	Actual utilization of foreign capital/GDP (%)	L3	Positive
	km/km^2^	L4	Positive
	Internet penetration rate (%)	L5	Positive
	Per capita education expenditure (yuan/person)	L6	Positive
	Proportion of research and development expenditure (%)	L7	Positive
Green development	Number of authorized green patents (pieces)	L8	Positive
	Green coverage rate of urban built-up areas (%)	L9	Positive
	Forest coverage rate (%)	L10	Positive
	Energy consumption elasticity coefficient	L11	Negative
Low-carbon development	Carbon productivity (10,000 yuan/ton)	L12	Negative
	Per capita energy consumption (ton of standard coal/person)	L13	Negative
	Carbon emission intensity per unit of GDP (billion yuan/10,000 tons)	L14	Negative
Circular development	Comprehensive utilization rate of industrial solid waste (%)	L15	Positive
	Proportion of coal consumption (%)	L16	Negative
	Reutilization rate of industrial water (%)	L17	Positive

To address concerns about the relevance of the “Proportion of coal consumption” indicator (L16) This study incorporates the “coal consumption ratio” indicator (L16) as a negative indicator into the dimension of circular development, reflecting its role in assessing the progress of sustainable resource utilization. Sustainable resource utilization is a core principle of the circular economy. A high proportion of coal consumption signifies reliance on non-renewable, high-emission fossil fuels, which contradicts the circular economy's focus on reducing resource dependency and minimizing environmental impact through efficient material flows and waste reduction. By measuring the reduction in coal consumption, this indicator captures the transition toward cleaner energy sources and improved energy efficiency, aligning with the “Reduce” principle of the circular economy's “Reduce-Reuse-Recycle” framework, as described in Section 2.1. This transition supports circular practices by decreasing the extraction of finite resources and reducing waste outputs, such as emissions from coal combustion, thereby contributing to the broader GLCCE objectives.

The Entropy-Weighted TOPSIS method combines entropy-based weighting with TOPSIS ranking to evaluate the GLCCE system. This hybrid approach first assigns weights to evaluation indicators using information entropy analysis, where entropy measures the uncertainty or variability in the data. Indicators with higher variability (i.e., more information) receive higher weights, as they are deemed more influential in distinguishing performance across years. The method then applies the TOPSIS to rank the weighted indicators against idealized benchmarks: the positive ideal solution (best possible values) and the negative ideal solution (worst possible values). This dual approach enables both granular indicator-level evaluation and a holistic composite assessment of the entire system.

Step 1: Dimensionless normalization of raw data


(1) $$j_{yn}=\{\begin{array}{l} \frac{i_{yn}-mini_{yn}}{maxi_{yn}-mini_{yn}}\times 0.999+0.001,i_{yn}\;is\;a\;positive\;indicator\\ \frac{maxi_{yn}-i_{yn}}{maxi_{yn}-mini_{yn}}\times 0.999+0.001,i_{yn}\;is\;a\;negative\;indicator \end{array}$$

Where, *i*_*yn*_(*y* = 1, 2, ⋯ , *w*; *n* = 1, 2, ⋯ , *t*) is the *y*-th indicator observation in year *n*, *j*_*yn*_ is the *y*-th indicator observation in year *n* after standardization. The addition of 0.001 after scaling to [0,1] is a standard adjustment in entropy-based methods to prevent zero values, which would lead to undefined logarithmic terms in the entropy formula. This small constant ensures all normalized values are positive, maintaining the integrity of the calculations.

Step 2: Calculate the information entropy *e*_*y*_ of the *y*-th indicator. First, find the *y*-th indicator probability distribution:


(2) $$\begin{array}{l} u_{yx}=\frac{J_{yn}}{\overset{t}{\underset{n=1}{\sum }}J_{yn}},y=1,2,\cdots \;,w \end{array}$$

Where *n*(*n* = 1, 2, ⋯ , *t*) is the length of the time series, the information entropy *e*_*y*_ of the *y*-th indicator is:


(3) $$\begin{array}{l} e_{y}=-\frac{1}{\ln\;t}\overset{t}{\underset{n=1}{\sum }}u_{y}\ln\;u_{yn} \end{array}$$

Step 3: Calculate the weight ω_*y*_ of the *y*-th indicator:


(4) $$\begin{array}{l} \omega_{y}=\frac{1-e_{y}}{\overset{w}{\underset{y=1}{\sum }}\left(1-e_{y}\right)} \end{array}$$

Step 4: Construct the canonical weighting matrix of the indicator:


(5) $$\begin{array}{l} K={\left(r_{yn}\right)}_{w\times t}={\left(\omega_{y}j_{y}\right)}_{w\times t\;} \end{array}$$

Step 5: Determine the optimal solution *S*^+^ and the worst solution *S*^−^:


(6) $$\begin{array}{l} S^{+}=\left(\max r_{1},\max r_{2},\max r_{3},\ldots ,\max r_{w}\right)=\left(S_{1}^{+},S_{2}^{+},\ldots ,S_{w}^{+}\right) \end{array}$$

(7) $$\begin{array}{l} S^{-}=\left(\min r_{1},\min r_{2},\min r_{3},\ldots ,\min r_{w}\right)=\left(S_{1}^{-},S_{2}^{-},\ldots ,S_{w}^{-}\right) \end{array}$$

Among them, *r*_*w*_ represents the normalized weighted value of the *w*-th indicator.

Step 6: Calculate the Euclidean distance between the weighted indicator observation *r*_*yn*_ and *S*^+^ and *S*^−^:


(8) $$\begin{array}{l} d_{n}^{+}=\sqrt{\overset{w}{\underset{y=1}{\sum }}{\left(r_{yn}-s_{n}^{+}\right)}^{2}}\left(1\leq n\leq t\right) \end{array}$$

(9) $$\begin{array}{l} d_{n}^{-}=\sqrt{\overset{w}{\underset{y=1}{\sum }}{\left(r_{yn}-s_{n}^{-}\right)}^{2}}\left(1\leq n\leq t\right) \end{array}$$

Step 7: Calculate the proximity *V*_*n*_:


(10) $$\begin{array}{l} V_{n}=\frac{d_{n}^{-}}{d_{n}^{+}+d_{n}^{-}},V_{n}\in \left[0,1\right] \end{array}$$

Where *V*_*n*_ is the score of the construction level of Chongqing's GLCCE system in year *n*. The bigger *V*_*n*_ is, the better the construction of the GLCCE system is in Chongqing. The larger *V*_*n*_ is, the better the construction of GLCCE system in Chongqing.

### 3.3 Panel regression model

The core explanatory variable, GIE, is calculated using a Data Envelopment Analysis (DEA) approach, a widely adopted method for assessing innovation efficiency. To reflect the role of open innovation, the DEA model incorporates input variables such as R&D expenditure, the number of R&D personnel, and the number of collaborative green R&D projects with external partners—capturing the collaborative R&D networks dimension of open innovation. Additionally, it includes the adoption of external green technologies, proxied by the number of green patents licensed or imported from outside Chongqing, representing external technology introduction. The output variables include the number of authorized green patents and the reduction in energy consumption per unit of GDP, which measure the tangible outcomes of these innovation efforts. GIE is not solely based on patent counts. Instead, it integrates multiple dimensions, such as collaborative R&D and energy efficiency improvements, ensuring a more comprehensive measure of innovation's effectiveness in driving sustainable outcomes. This approach aligns with Chongqing's industrial and ecological context, where the adoption and efficiency of green technologies are critical for GLCCE success.

While environmental quality is a multifaceted concept encompassing various pollutants and ecological indicators, this study focuses on SO_2_ concentration as the primary measure of air pollution for several reasons. SO_2_ is a critical indicator of industrial emissions and has been widely used in environmental economics and policy studies to assess air quality, particularly in regions with heavy industrial activity Boni et al. (31). In China, SO_2_ has historically been a key target for air pollution control due to its significant health impacts and its role as a precursor to acid rain and particulate matter (Li et al.) (32). Moreover, SO_2_ concentration is highly correlated with other pollutants such as PM2.5 and NO_2_, making it a representative proxy for overall air pollution levels (Cai et al.) (33). In the context of Chongqing Municipality, SO_2_ is particularly relevant due to the city's industrial structure, which includes energy-intensive sectors such as steel and chemical manufacturing. For instance, Chongqing's Action Plan for Air Pollution Prevention and Control (2018–2022) specifically targets SO_2_ reduction as a priority, reflecting its ongoing significance in local environmental policy.

Based on the analysis of influencing factors, this study uses SO_2_ concentration as the dependent variable to measure environmental quality. The explanatory variables include GIE with undesirable outputs, transportation accessibility (ACC), openness to external trade (EXT), economic development level (EDL), information technology level (ITL), industrial structure (INS), urban cultural level (CUL) and research and development investment (RDI). A fixed-effects panel regression model is constructed to measure the impact of GIE on environmental quality while controlling for socioeconomic factors. The equation is as follows:


(11) $$\begin{array}{l} SO_{2}=\\ \begin{array}{c} \alpha_{0}+\alpha_{1}GIE+\alpha_{2}\;ACC\;+\alpha_{3}EXT+\alpha_{4}EDL\\ +\alpha_{5}ITL+\alpha_{6}CUL+\alpha_{7}RDI+\;Year_{x}+\;City_{n}+\varepsilon_{xn} \end{array} \end{array}$$

Equation 11 is a panel regression model controlling economic and social factors. SO_2_ is the concentration of SO_2_ in the atmosphere of Chongqing. α_0_ is a constant term; α_1_ to α_6_ are the coefficients to be estimated; and α_*xn*_ is a random error term. The core explanatory variable is GIE, and transportation accessibility (ACC), etc. are its economic and social factors control variables.

In the model, the coefficient α_1_ corresponds to the explanatory variable of primary interest, focusing on how GIE affects environmental quality in Chongqing while controlling for other variables. If α_1_ is negative, it indicates that GIE can promote the improvement of atmospheric quality in Chongqing, and the higher the GIE, the better the atmospheric quality. If α_1_ is positive, it means that the higher the GIE, the more it hinders the improvement of atmospheric quality.

### 3.4 Models of mediating effects

The study of mediating effect pathways primarily examines how explanatory variables (*I*) indirectly influence explained variables (*J*) through one or more mediating variables (*W*). By revealing the non-direct pathways through which explanatory variables affect outcome variables, this analytical approach provides deeper insights into the formation mechanisms of variable interactions. In academic research, the causal stepwise regression method is widely employed for mediating effect testing (34). It verifies mediating effects by constructing three regression equations with distinct causal logic. The specific model framework is as follows:


(12) $$\begin{array}{l} J=cI+\varepsilon_{1} \end{array}$$

(13) $$\begin{array}{l} W=gI+\varepsilon_{2} \end{array}$$

(14) $$\begin{array}{l} J=c^{\prime }I+hW+\varepsilon_{3} \end{array}$$

In the first step, the regression coefficient c is tested for significance according to Equation 12 to determine whether there is a relationship between *I* and *J*. If *c* is significant, it means that *I* will have an effect on *J*, so that the next regression can be carried out.

The second step is to test the significant level of the regression coefficient a according to Equation 13. Based on the result of *g* determine whether there will be an effect on *W*. If the result shows that *g* is significant, then it means that *I* will have an effect on *W*, and then the third step of the mediating effect test can be carried out.

**Figure 1:**
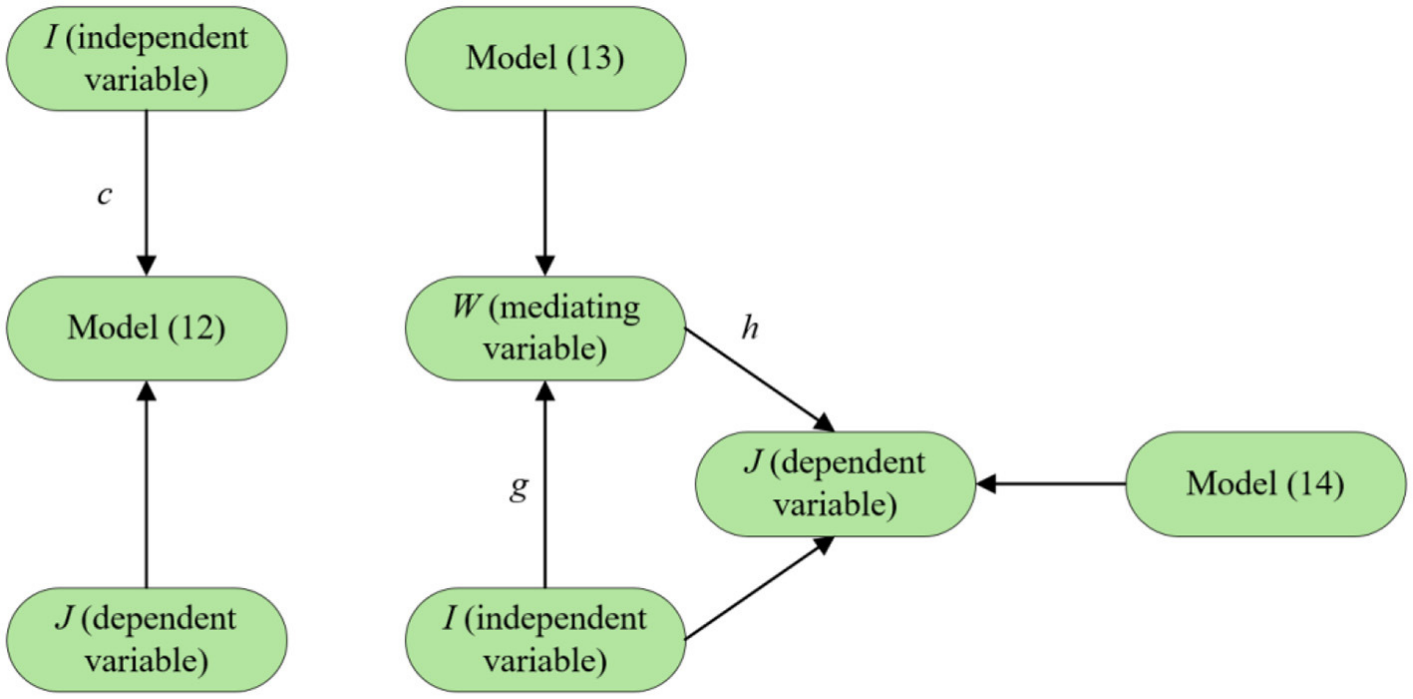
Flow chart of mediating effect.

**Figure 2:**
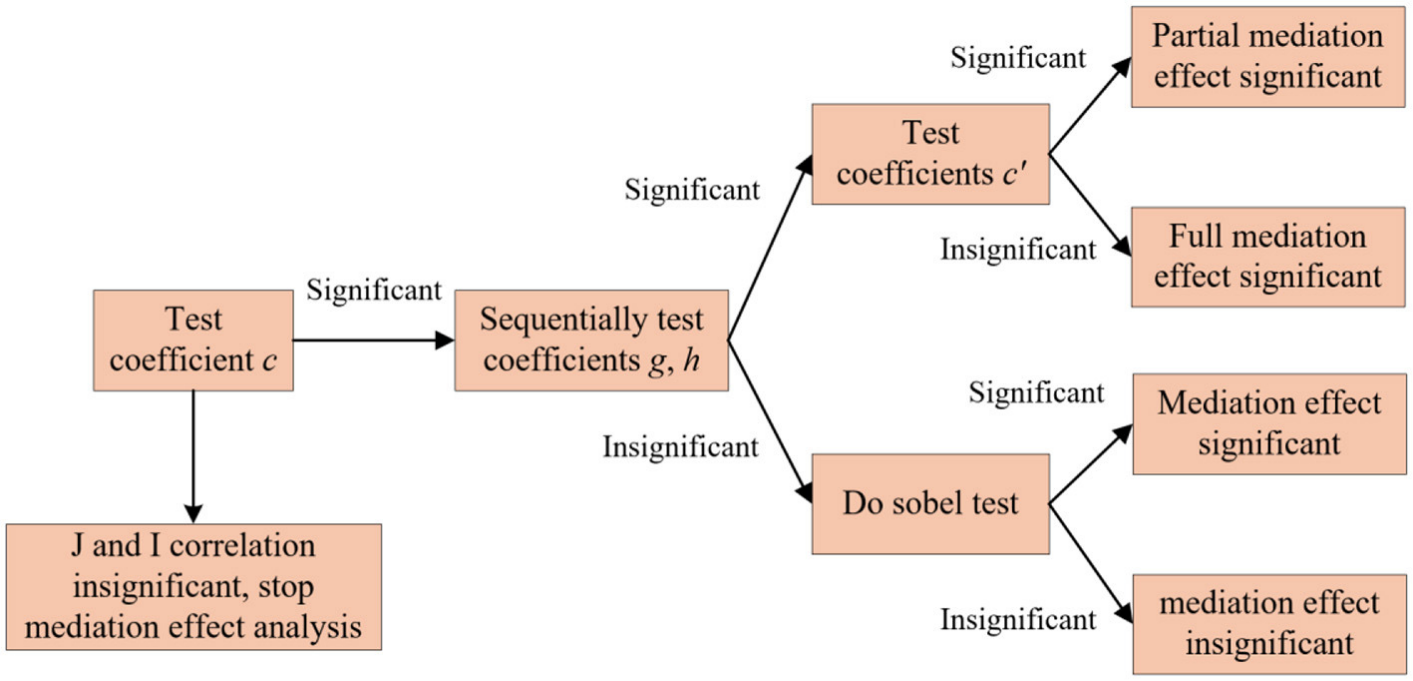
Mediating effect transmission mechanism.

In the third step, the regression coefficients *c*′ and *h* are tested according to Equation 14 to determine the relationship between *I, W*, and *J* to determine the type of mediating effect produced. If the coefficient *h* and coefficient *c*′ are both significant, it means that there is a partially mediating effect is significant, that is, *I* not only directly affects *J*, but also can indirectly affect *J* by affecting *W*. If the coefficient *h* is significant and the coefficient *c*′ is not significant, then there is a full mediating effect. That is, *I* has an effect on *J* through *W*. If the coefficient *h* is not significant, then the Sobel test should be carried out. This test helps determine whether the mediating effect is significant or not, based on the significance of its results. The operation and result analysis path are shown in Figures 1, 2, respectively.

This paper constructs the following mediating effect model:


(15) $$\begin{array}{c} SO_{2}=\alpha_{0}GIE+\beta_{1}\;ACC\;+\beta_{2}EXT+\beta_{3}EDL+\beta_{4}\;ITL\\ +\beta_{5}CUL+\beta_{6}RDI{+\;Year\;}_{x}+{\;City\;}_{n}+\varepsilon_{xn} \end{array}$$

(16) $$\begin{array}{c} INS=\alpha_{1}GIE+\beta_{1}\;ACC\;+\beta_{2}EXT+\beta_{3}EDL+\beta_{4}\;ITL\\ +\beta_{5}CUL+\beta_{6}RDI{+\;Year\;}_{x}+{\;City\;}_{n}+\varepsilon_{xn} \end{array}$$

(17) $$\begin{array}{c} SO_{2}=\alpha_{2}GIE+\alpha_{3}INS+\beta_{1}\;ACC\;+\beta_{2}EXT+\beta_{3}EDL\\ +\beta_{4}\;ITL+\beta_{5}CUL+\beta_{6}RDI{+\;Year\;}_{x}+{\;City\;}_{n}+\varepsilon_{xn} \end{array}$$

The coefficients β_*x*_ in Equations 15–17 above represent the regression results of the control variables. α_0_ represents the total effect of GIE on the improvement of environmental quality without the introduction of mediator variables. α_1_ represents the level of the effect of the solution variable GIE on the mediator variable, i.e., the industrial structure. α_1_ is significant, indicating that there is an indirect effect of the GIE on the industrial structure. The next step of the test analysis. α_2_ and α_3_ coefficients are significant indicating that there is a part of the mediating effect is significant. This implies that GIE not only affects environmental quality through the mediating variable of industrial structure, but also affects environmental quality by itself. The secondary industry is generally considered to be the industry that consumes resources and produces serious environmental pollution. The tertiary industry, on the other hand, is the one that includes computer services, high technology and other less polluting enterprises. China's industrial structure pursues the development of the tertiary industry, so this paper selects the ratio of the tertiary industry to the secondary industry to characterize the optimization and upgrading of the INS as a mediating variable to analyze the indirect impact mechanism of GIE.

## 4 Result analysis and discussion

### 4.1 Comprehensive evaluation analysis of the construction level of GLCCE system

Using the statistical software R4.1.1, the weights of the evaluation indicators are determined according to the calculation steps of the entropy weighting method, i.e., Equations 1, 2. Then, the corresponding second-level indicator weights are summed to get the first-level indicator weights, as shown in Table 2.

**Table 2:** Indicator weights results.

**Primary indicators**	**Secondary indicators**	**Indicator weights**	**Primary indicator weights**
Economic and social development	Per capita GDP (10,000 yuan)	0.034	0.297
	Proportion of the tertiary industry (%)	0.044	
	Actual utilization of foreign capital/GDP (%)	0.043	
	km/km^2^	0.038	
	Internet penetration rate (households per 100 people)	0.032	
	Per capita education expenditure (yuan/person)	0.051	
	Proportion of research and development expenditure (%)	0.055	
Green development	Number of authorized green patents (pieces)	0.047	0.222
	Green coverage rate of urban built-up areas (%)	0.062	
	Forest coverage rate (%)	0.068	
	Energy consumption elasticity coefficient	0.045	
Low-carbon development	Carbon productivity (10,000 yuan/ton)	0.069	0.204
	Per capita energy consumption (ton of standard coal/person)	0.067	
	Carbon emission intensity per unit of GDP (billion yuan/10,000 tons)	0.068	
Circular development	Comprehensive utilization rate of industrial solid waste (%)	0.072	0.201
	Proportion of coal consumption (%)	0.063	
	Reutilization rate of industrial water (%)	0.066	

The measurement indicator sequence that affects the construction level of Chongqing's GLCCE system is: economic and social development > green development > low-carbon development > circular development. The weights of each secondary indicator are all above 0.2, indicating that the contributions of each subsystem to GLCCE development are relatively balanced. The experimental results show that promoting GLCCE development requires coordinated efforts from all aspects to further achieve high-quality development of a GLCCE and society.

**Figure 3:**
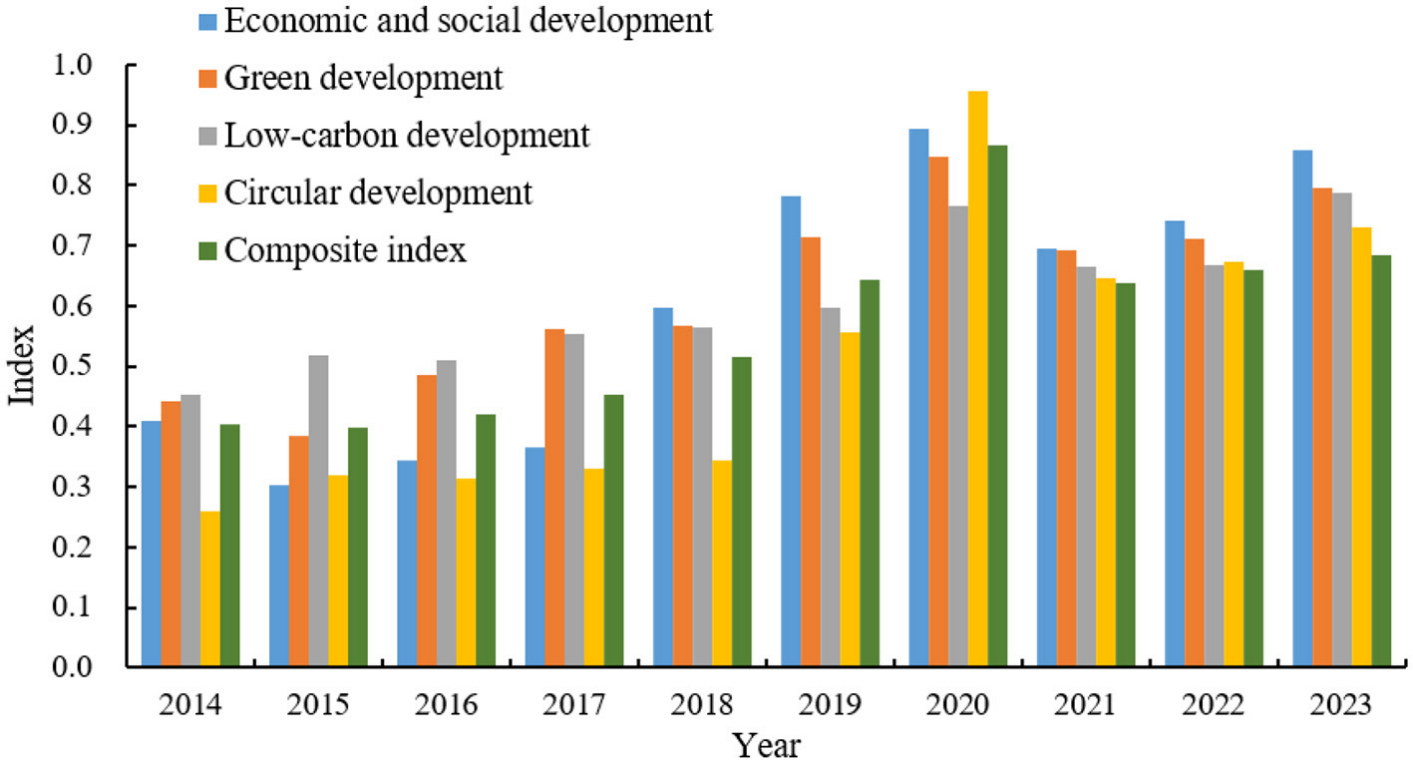
Index of the construction level of Chongqing's GLCCE system.

**Figure 4:**
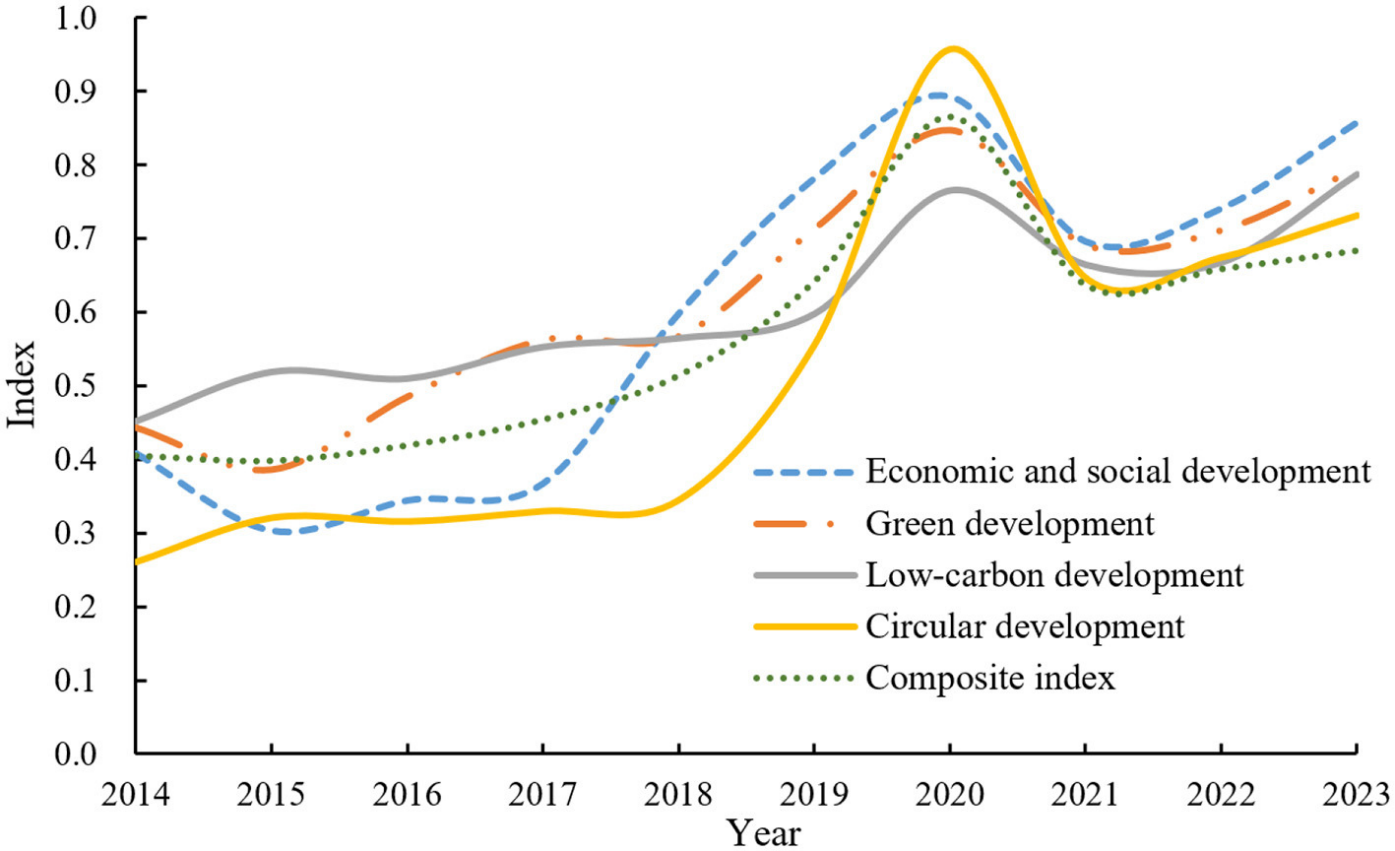
Trend of the construction level of Chongqing's GLCCE system.

Based on the standardized data and calculated indicator weights, the TOPSIS method, namely Equations 3–10, was used to program and calculate the construction levels and comprehensive index of each subsystem of Chongqing's GLCCE system from 2014 to 2023 in the statistical software R4.1.1. The results are shown in Figure 3. Then, the index results in Figure 3 were plotted as the trend chart of the construction level of Chongqing's GLCCE system, as shown in Figure 4.

From Figures 3, 4, it can be observed that from 2014 to 2023, Chongqing's GLCCE development composite index showed a fluctuating trend of “rise, adjustment, and stabilization,” increasing from 0.405 to 0.684, with an average annual growth rate of 4.8%. The period 2014–2017 marked steady growth (0.405–0.454). Accelerated progress occurred from 2018 to 2020, driven by the “Yangtze River Economic Belt Green Development” policy, peaking at 0.866 in 2020 (a 90.7% increase from 2017). After 2021, external shocks such as global energy crises and domestic industrial restructuring contributed to a decline in the composite index to 0.684 by 2023, still 68.9% higher than the 2014 baseline. By dimension: economic and social development rose most sharply, from 0.408 to 0.858, peaking at 0.893 in 2020. Green development improved steadily (0.443–0.795), with urban ecological governance achieving a peak of 0.847 in 2020. Low-carbon development fluctuated notably, surging to 0.766 in 2020 before stabilizing at 0.788 in 2023. Circular development spiked to 0.957 in 2020 due to breakthroughs in industrial waste utilization, then adjusted to 0.731 by 2023 amid production reforms. The data highlight strong synergy across dimensions during policy-driven growth (2018–2020), while circular economy resilience became the stabilizing pillar under external shocks (2021–2023).

### 4.2 Analysis of regression results

Based on the panel data of SO_2_ concentration in Chongqing Municipality from 2014 to 2023, this paper empirically examines the influencing factors of GLCCE and sustainable development. The study adopts the panel regression model and uses Stata16.0 software to conduct stepwise regression analysis of relevant factors. The specific analysis results are shown in Table 3, in which column 7 is the benchmark regression results for focusing on the impact of GIE on the GLCCE and sustainable development of Chongqing Municipality.

**Table 3:** Results of environmental spillover effect of GIE under the control of economic and social factors.

**Explanatory variable**	**1**	**2**	**3**	**4**	**5**	**6**	**7**
GIE	−0.271^**^ (−2.01)	−0.205 (−1.32)	−0.295^*^ (−1.73)	−0.242^***^ (−2.82)	−0.238^***^ (−2.96)	−0.210^**^ (−2.64)	−0.218^**^ (−2.60)
ACC	—	−0.228 (−1.37)	−0.291 (−1.66)	−0.14 (−1.25)	−0.224^*^ (−1.94)	−0.234^*^ (−1.93)	−0.241^*^ (−1.96)
EXT	—	—	1.205 (−1.64)	4.503^***^ (−8.93)	3.785^***^ (−7.47)	3.921^***^ (−7.44)	3.950^***^ (−7.45)
EDL	—	—	—	−1.987^***^ (−24.89)	−1.405^***^ (−9.21)	−1.365^***^ (−9.23)	−1.392^***^ (−8.98)
ITL	—	—	—	—	−0.308^***^ (−4.32)	−0.124^**^ (−2.30)	−0.131^**^ (−2.38)
CUL	—	—	—	—	—	−0.281^***^ (−3.98)	−0.289^***^ (−3.96)
RDI	—	—	—	—	—	—	−0.176^**^ (−2.59)
Year	Control	Control	Control	Control	Control	Control	Control
City	Control	Control	Control	Control	Control	Control	Control
Constant	3.128^***^ (−28.44)	4.115^***^ (−5.41)	4.420^***^ (−5.56)	24.65^***^ (−28.43)	20.72^***^ (−17.02)	21.03^***^ (−17.24)	21.25^***^ (−17.21)
*R* ^2^	0.03	0.505	0.532	0.878	0.885	0.875	0.882
*N*	166	166	166	166	166	166	166
Hausman	0.3012	0	0	0.0008	0	0.0002	0.0001

^*^p < 0.1, ^**^p < 0.05, ^***^p < 0.01; t-statistics in parentheses, N is sample size.

From the results of Hausman test in Table 3, it can be seen that the panel fixed effects model is suitable for analyzing the environmental spillover effects of GIE in Chongqing. The specific conclusions are as follows:

The regression analysis reveals a significant relationship between GIE and environmental quality, as measured by SO_2_ concentration, a key indicator within the GLCCE framework. Specifically, the coefficient for GIE is −0.218 (*t* = −2.60, *p* < 0.05), this indicates that a one-unit increase in GIE, on average, reduces SO_2_ concentration by 0.218 units. The statistical significance at the 5% level and the negative coefficient highlight GIE's pivotal role in improving environmental quality, a core objective of the GLCCE. Economically, this suggests that policies enhancing GIE, such as Chongqing's “Green Technology R&D Center” and the “Industrial Desulfurization Technology” (reducing SO_2_ emissions by 25% in key enterprises in 2023), can yield tangible environmental benefits, supporting sustainable urban development in Western China.

To further elucidate the impact of GIE on GLCCE, this paper emphasize that GIE serves as a critical driver of the green low-carbon circular economy by enhancing resource efficiency, reducing environmental pollution, and promoting sustainable industrial practices. Specifically, GIE facilitates the efficient use of resources through innovations like the “Industrial Desulfurization Technology,” which directly lowers SO_2_ emissions—a key GLCCE indicator—as evidenced by the regression coefficient of −0.218 in Table 3. This aligns with the circular economy principles outlined in Section 2.1, such as minimizing waste and optimizing resource cycles. Additionally, the mediating effect of INS reveals that GIE indirectly bolsters GLCCE by supporting a shift toward less resource-intensive industries (e.g., a 1% increase in GIE correlates with a 0.123% increase in INS, reducing SO_2_ concentration further). These combined effects highlight GIE's pivotal role in advancing GLCCE through both direct environmental improvements and structural economic transformation.

Further analysis of additional variables complements these findings by providing more insights into the multifaceted nature of GLCCE. The coefficient for transportation accessibility (ACC) is −0.241 (*t* = −1.96, *p* < 0.1), suggesting a modest reduction in SO_2_ concentration with improved infrastructure, though the wider interval reflects less precision. In contrast, the coefficient for openness to external trade (EXT) is 3.950 (*t* = 7.45, *p* < 0.01), indicating that foreign investment significantly increases pollution, a challenge for GLCCE implementation. This positive effect underscores the need for stricter environmental oversight of foreign-funded projects. Finally, the coefficient for economic development level (EDL) is −1.392 (*t* = −8.98, *p* < 0.01), demonstrating that green economic growth substantially lowers SO_2_ concentration, aligning economic progress with environmental goals. For instance, Chongqing's shift to high-tech industries and ultra-low emission standards reduced SO_2_ emissions by 15,000 tons, illustrating GLCCE's potential to harmonize development and sustainability.

The coefficient for ITL is −0.131 (*t* = −2.38, *p* < 0.05), indicating that digitization technology effectively suppresses pollution. Chongqing Municipality's “environmental protection big data platform” has access to more than 600 enterprises, and 53 incidents of excessive emissions were intercepted through real-time monitoring in 2023. A smart manufacturing enterprise optimized energy consumption through AI, and reduced SO_2_ emission intensity by 18%, reflecting the technology-driven environmental benefits.

The coefficient for CUL is−0.289 (*t* = −3.96, *p* < 0.01), reflecting the improvement of citizens' awareness of environmental protection to promote low-carbon development. 90% of Chongqing's “green campuses” will be covered by 2023, and the number of community environmental protection volunteers will increase to 500,000 to promote the participation rate of garbage classification exceeding 80%, thus reducing pollution at the source of lifestyles. Reduce pollution at the source.

The coefficient for RDI is −0.176 (*t* = −2.59, *p* < 0.05), showing that for every 1% increase in R&D investment, SO_2_ concentration decreases by 17.6%. Chongqing Municipality will invest more than 12 billion RMB in clean technology R&D in 2023, and the application of carbon capture technology in power plants will reduce SO_2_ emissions by 30% per unit. The nanocatalytic desulfurization material developed by a university was awarded a national patent, further strengthening the role of innovation in supporting environmental protection. Although RDI exhibits a relatively smaller marginal coefficient (−0.176) on SO_2_ reduction compared to EDL (−1.392), it should not be undervalued in policy formulation. This is because RDI contributes not only directly to pollution control technologies, such as the deployment of carbon capture and desulfurization innovations, but also indirectly enhances GIE, which in turn improves both environmental quality and industrial structure transformation. Moreover, the mediating effect analysis confirms that RDI significantly boosts INS, a variable proven to reduce pollution through industrial upgrading. RDI remains a critical enabler of long-term GLCCE objectives by fostering innovations that enhance resource efficiency and support sustainable practices. RDI-driven advancements, such as carbon capture technology in power plants, achieved a 30% per-unit reduction in SO_2_ emissions.

Table 4 shows the regression results of the mediating effect of INS between GIE and GLCCE and sustainable development, revealing the direct and indirect influence paths.

**Table 4:** Mediating effect regression results.

**Explanatory variable**	**Model 1: SO_2_**	**Model 2: INS**	**Model 3: SO_2_**
GIE	−0.212^**^ (0.0786)	0.123^**^ (0.0517)	−0.185^**^ (0.0782)
INS			−0.257^**^ (0.108)
ACC	−0.218^*^ (0.126)	0.0688 (0.0695)	−0.215^*^ (0.119)
EXT	3.754^***^ (0.523)	0.0776 (0.336)	3.742^***^ (0.521)
EDL	−1.345^***^ (0.149)	0.234^***^ (0.0978)	−1.285^***^ (0.154)
ITL	−0.120^**^ (0.0528)	0.164^***^ (0.0349)	−0.209^***^ (0.0771)
CLU	−0.283^***^ (0.0704)	0.206^***^ (0.0478)	−0.214^***^ (0.0740)
RDI	−0.296^***^ (0.0715)	0.215^***^ (0.0463)	−0.229^***^ (0.0746)
Constant	21.95^***^ (1.201)	−5.282^***^ (0.811)	20.48^***^ (1.354)
Year	Control	Control	Control
City	Control	Control	Control
*R* ^2^	0.869	0.878	0.803
*N*	166	166	166

^*^p < 0.1, ^**^p < 0.05, ^***^p < 0.01.

According to the regression results in Table 4, it can be seen that there is a mediating effect of INS. This indicates that GIE can influence the GLCCE and sustainable development through INS. In addition, the effect of GIE on environmental quality is still significant after adding the mediator variable, which indicates that the mediator effect is incomplete mediator effect, i.e., GIE not only affects the environmental quality by itself, but also produces the effect indirectly through INS. From the model (2), GIE has a significant positive effect on INS at the 5% level, indicating that an increase in GIE is beneficial to INS; a 1% increase in GIE promotes a 0.123% increase in INS. EDL, ITL, and CUL, as well as RDI have a stronger effect on INS, and all of them have significant impacts at the 1% level, but with different degrees of impacts; the degree of impacts is 0.234 for EDL, 0.164 for ITL, 0.206 for CUL and 0.215 for RDI. Putting INS into the regression model, according to the results of model (3), it can been found that INS is beneficial to the improvement of environmental quality, and its impact on environmental quality is significant at 5% level. The optimization and upgrading of industrial structure can gradually reduce the dependence on traditional production factors such as energy, realize the intensive use of energy, and then reduce pollution emissions, reduce the degree of air pollution, and realize the GLCCE and sustainable development. Furthermore, regional differences in industrial composition lead to varying strengths of the mediating effect of INS across Chongqing. In particular, the Main Urban Area—dominated by high-tech industries and advanced services—exhibits a significantly stronger mediating effect of INS compared to the Wuling Mountain Area, where traditional industries such as agriculture, mining, and basic manufacturing still prevail. In the Main Urban Area, green innovation efficiency (GIE) more effectively drives the upgrading of industrial structure due to the stronger presence of innovation infrastructure, R&D institutions, and talent clusters. This structural transformation amplifies the impact of GIE on environmental quality through more efficient energy usage and lower emission intensity. In contrast, in the Wuling Mountain Area, the industrial base is less flexible, and the adoption of green technologies tends to lag behind, thereby weakening the mediating channel of INS. These findings underscore the need for differentiated policy interventions that account for local industrial characteristics. For example, in regions like Wuling, enhancing green infrastructure and vocational retraining could strengthen the transmission of GIE into sustainable development outcomes.

Avoiding unexpected errors in regression results based on specific samples and ensuring the reliability of the assessment results. This paper adopts the robustness test of excluding abnormal years and adding control variables.

(1) Excluding the abnormal year: in the sample period selected in this paper, the abnormalities of the GIE value in 2020 caused by epidemics are more obvious, which may lead to the inaccuracy of the environmental effect of GIE. Therefore, this anomalous year is excluded, and a new sample group is constructed to re-regress and test whether the regression results are universal.
(2) Increase the control variables: the number of population has a close relationship with the environment, and with the increase of population density in the region, the consumption of all kinds of resources in the region will also increase. Consumption of resources to a large extent will have an impact on the diverse ecosystems in the region, so this paper increases the population density as a control variable for robustness testing. The relevant regression results are shown in Table 5.

**Table 5:** Robustness test results.

**Explanatory variable**	**Main regression model (baseline)**	**After excluding the 2020 sample**	**After adding the population density control variable**
GIE	−0.204^**^ (−2.64)	−0.218^**^ (−2.55)	−0.197^**^ (−2.38)
ACC	−0.227^*^ (−1.93)	−0.241^*^ (−1.89)	−0.235^*^ (−1.85)
EXT	3.843^***^ (−7.44)	3.950^***^ (−7.45)	3.821^***^ (−7.32)
EDL	−1.337^***^ (−9.23)	−1.392^***^ (−8.98)	−1.305^***^ (−8.75)
ITL	−0.119^**^ (−2.30)	−0.131^**^ (−2.38)	−0.125^**^ (−2.20)
CLU	−0.272^***^ (−3.98)	−0.289^***^ (−3.96)	−0.265^***^ (−3.85)
RDI	−0.176^**^ (−2.59)	−0.189^**^ (−2.48)	−0.163^**^ (−2.30)
POP			−0.148^**^ (−2.45)
Year	Control	Control	Control
City	Control	Control	Control
Constant	20.84^***^ (−17.24)	21.25^***^ (−17.21)	19.72^***^ (−16.85)
*R* ^2^	0.871	0.882	0.875
*N*	166	156	166

^*^p < 0.1, ^**^p < 0.05, ^***^p < 0.01.

As can be seen from Table 5, after excluding the 2020 data, the GIE fluctuates slightly from −0.204 to −0.218, which still passes the 5% significance test (*t* = −2.55). This indicates that outliers due to epidemics did not significantly affect the core findings. For example, Chongqing Municipality experienced an anomalous 8% decrease in SO_2_ concentration in 2020 due to a short period of stagnation of industrial activities as a result of the anti-epidemic policy, but the model still showed a sustained effect of the GIE on emission reduction after the exclusion. *r*^2^ increased from 0.871 to 0.882, suggesting that the model's explanatory power was enhanced by the exclusion of the anomalous years. The POP coefficient was −0.148 (*t* = −2.45, *p* < 0.05), suggesting that for every 1% increase in population density, the SO_2_ concentration decreased by−0.148. The POP coefficient was −0.148 (*t* = −2.45, *p* < 0.05), indicating that for every 1% increase in population density, SO_2_ concentration decreased by 14.8%. This is related to the strategy of “new urbanization” in Chongqing, where the concentration of population in the central urban area promotes the intensification of public services and the reduction of dispersed sources of pollution. The coefficients of the GIE and EDL variables are < 5% different from those of the main regression, and the significance level remains unchanged. For example, the coefficient of EDL is slightly adjusted from −1.337 to −1.305 (*t* = −8.75), which still passes the 1% test, confirming the synergistic effect of economic growth and environmental protection policies. The coefficient of RDI rises slightly to −0.189 (*t* = −2.48), and the significance level stays at 5%, which suggests that outliers due to epidemics have not weakened the environmental benefits of RDI. In addition, *R*^2^ stabilized at 0.875, and the added variables did not lead to overfitting, indicating a reliable model structure.

## 5 Conclusion

This study explores the role of GIE in promoting GLCCE and sustainable urban development in Chongqing Municipality as an example. By constructing the evaluation index system and using the entropy weight-TOPSIS model and panel regression model, the study finds that: (1) The significant impact of GIE: GIE significantly enhances the development level of GLCCE in Chongqing and promotes the improvement of the efficiency of the circular economy through technological progress and optimal allocation of resources. Specifically, for every 1% increase in GIE, the SO_2_ concentration in Chongqing Municipality is reduced by 21.8%. This suggests that GIE is an important driver for achieving environmental quality improvement and sustainable development. (2) Mediating role of INS: GIE indirectly affects environmental quality through optimizing industrial structure. It is found that the improvement of GIE promotes the development of tertiary industry, while the expansion of tertiary industry helps to reduce the reliance on traditional energy-intensive production factors, thus reducing pollution emissions and improving air quality. (3) Robustness test results: Through the robustness test with the exclusion of abnormal years and the addition of control variables, the results of the study remain consistent, further verifying the positive impact of GIE on environmental quality.

The findings highlight Chongqing's progress in balancing ecological protection and industrial transformation under its unique geographical and policy conditions. However, cities in western China exhibit significant heterogeneity. For instance, provinces like Shaanxi (characterized by arid climates and energy-driven economies) or Sichuan (with agricultural dominance and hydropower reliance) face distinct challenges in GLCCE implementation. These regions may require differentiated strategies that account for local resource availability, industrial legacies, and policy frameworks. Future studies would integrate regional variables such as topography, energy mix, and fiscal decentralization to evaluate the transferability of Chongqing's GIE-driven model across diverse contexts. Moreover, future research will expand this analysis by incorporating additional indicators such as PM2.5, CO_2_, or water quality metrics to provide a more comprehensive assessment.

## Data Availability

The original contributions presented in the study are included in the article/supplementary material, further inquiries can be directed to the corresponding author.
